# Early Steps in Autophagy Depend on Direct Phosphorylation of Atg9 by the Atg1 Kinase

**DOI:** 10.1016/j.molcel.2014.01.024

**Published:** 2014-02-06

**Authors:** Daniel Papinski, Martina Schuschnig, Wolfgang Reiter, Larissa Wilhelm, Christopher A. Barnes, Alessio Maiolica, Isabella Hansmann, Thaddaeus Pfaffenwimmer, Monika Kijanska, Ingrid Stoffel, Sung Sik Lee, Andrea Brezovich, Jane Hua Lou, Benjamin E. Turk, Ruedi Aebersold, Gustav Ammerer, Matthias Peter, Claudine Kraft

(Molecular Cell *53*, 471–483)

In the Online Now version of this paper published ahead of print, the name of author Alessio Maiolica was misspelled. This name now appears correctly in both the print and online versions of this paper. The authors apologize for any inconvenience this has caused.

